# Combined AC‐electrokinetic effects: Theoretical considerations on a three‐axial ellipsoidal model

**DOI:** 10.1002/elps.201800015

**Published:** 2018-03-30

**Authors:** Jan Gimsa

**Affiliations:** ^1^ Department of Biophysics University of Rostock Rostock Germany

**Keywords:** AC electrokinetic fingerprinting, Broken spectra, Characteristic equations, Chicken‐red blood cells, Circular field orientation

## Abstract

AC fields induce charges at the structural interfaces of particles or biological cells. The interaction of these charges with the field generates frequency‐dependent forces that are the basis for AC‐electrokinetic effects such as dielectrophoresis (DEP), electrorotation (ROT), electro‐orientation, and electro‐deformation. The effects can be used for the manipulation or dielectric single‐particle spectroscopy. The observation of a particular effect depends on the spatial and temporal field distributions, as well as on the shape and the dielectric and viscoelastic properties of the object. Because the effects are not mutually independent, combined frequency spectra are obtained, for example, discontinuous DEP and ROT spectra with ranges separated by the reorientation of nonspherical objects in the linearly and circularly polarized DEP and ROT fields, respectively. As an example, the AC electrokinetic behavior of a three‐axial ellipsoidal single‐shell model with the geometry of chicken‐red blood cells is considered. The geometric and electric problems were separated using the influential‐radius approach. The obtained finite‐element model can be electrically interpreted by an RC model leading to an expression for the Clausius–Mossotti factor, which permits the derivation of force, torque, and orientation spectra, as well as of equations for the critical frequencies and force plateaus in DEP and of the characteristic frequencies and peak heights in ROT. Expressions for the orientation in linearly and circularly polarized fields, as well as for the reorientation frequencies were also derived. The considerations suggested that the simultaneous registration of various AC‐electrokinetic spectra is a step towards the dielectric fingerprinting of single objects.

**Abbreviations**
CFO: circular field orientation
CRBC: chicken‐red blood cell
DEP: dielectrophoresis
LFO: linear field orientation
ROT: electrorotation

## 1 Introduction

For dielectric single‐particle spectroscopy, a variety of AC‐electrokinetic force effects can be employed as an alternative to impedance measurements 1, 2, 3. Even though impedance micro‐chambers for single biological cells have been developed 4, 5, 6, 7, 8, 9, 10, 11, 12, it can be shown that AC‐electrokinetic spectra have a higher resolution for the properties of freely suspended individual objects with the trade‐off of a higher field strength having to be employed in order to induce detectable movements 3. The movements can also be used for the separation of cells according to their properties 7, 11, 12, 13, 14, 15. Moreover, AC‐electrokinetic force effects act on “smeared interfaces”, which may be generated by thermal gradients 3, 7, 16.

As early as the 1960s, the spinning of biological cells was described in dielectrophoresis (DEP) experiments, which suggested that DEP and electrorotation (ROT) are somehow interrelated 17, 18. The first correct explanation for the rotational effect of biological cells was given by Holzapfel et al. 19. Arnold and Zimmermann 20 were the first biologists to use a rotating electric field after an idea of H.P. Schwan (personal information). At that time, biologists were not aware that considerable work had already been performed in the field of physics 21, 22.

Nowadays, the frequency dependence of translations and rotations of single cells is microscopically analyzed in inhomogeneous (DEP) and rotating (ROT) external fields, respectively 2, 6, 7, 23, 24, 25, 26, 27, 28, 29. From the DEP and ROT spectra, dielectric properties 30, 31, 32 and cell physiological properties 8, 14, 33, 34, 35, 36, 37 can be recalculated applying appropriate cell models. Such as for impedance, multi‐shell spherical, cylindrical and ellipsoidal models are readily available 2, 31, 38, 39, 40. In the low frequency range, different processes may influence AC‐electrokinetic measurements, as electrode polarizations and hydrodynamic relaxations of electro‐osmotically induced convections (see: 27 and references cited therein). Below 100 Hz in particular, the suspension medium can no longer be considered a fixed reference system. This, and difficulties in the application of frequencies above the high MHz range, are the reasons that the so‐called ß‐dispersions in the medium frequency range are best characterized. For biological cells, the main components of the ß‐dispersions are the structural Maxwell–Wagner‐dispersions, i.e. the dispersions of polarizations of membrane systems, as well as the dispersions of the bulk volume polarizations according to their different conductivities 3, 41, 42, 43. Beyond their dispersions, membrane systems are capacitively bridged, while the bulk media are polarized according to their different permittivities.

It is interesting to note that the same polarization models are applied beyond the fields of colloid sciences and biotechnology, for example, for the meteorological problem of atmospheric dust particles covered by a water layer 44. Here, the same Laplace solution for the polarization of a single‐shell ellipsoid, the standard model of a biological cell, had already been derived before the biological work 40, 45, 46.

One motivation for this paper is to review the strong interrelations of diverse dielectric effects. In former experiments, we used microstructure chambers to investigate DEP, ROT, orientation, and the collection of three‐axial chicken‐red blood cells (CRBCs) in linear and circular fields 47, 48. This paper presents a unified theoretical description of the effects that had been observed with CRBCs. Detailed experimental results will be presented in a following manuscript.

## 2 AC electrokinetic effects

### 2.1 The interrelation of DEP and ROT spectra

**Figure 1:**
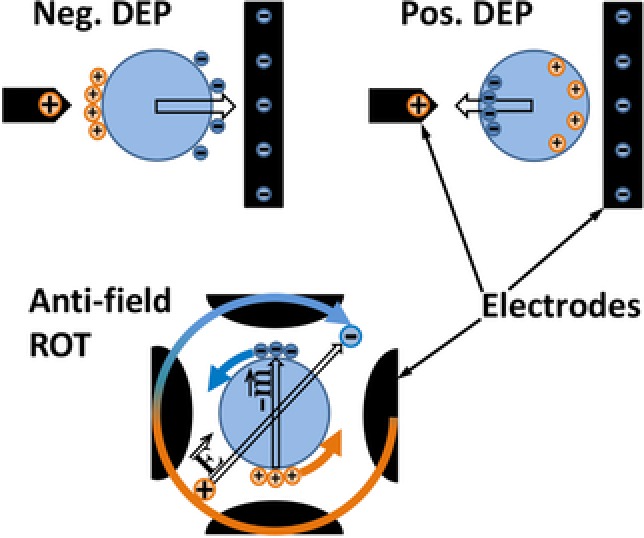
Sketch illustrating force and torque generation in DEP and ROT. Top: Snapshot of the charge distribution for the negative (left: effective polarizability of object lower than suspension medium) and the positive (right: effective polarizability of object higher than suspension medium) plateaus and the resulting effective force directions (arrows). Bottom: Snapshot of the external field vector and the induced dipole moment in the membrane dispersion frequency range mediating the transition from the negative to the positive DEP plateaus. While the field and the dipole moment rotate clockwise at the external field frequency the torque, resulting from their interaction induces a much slower counter‐clockwise (anti‐field) rotation.

The polarization and dielectric behavior of biological cells can qualitatively be described by shelled models. Figure 1 illustrates the interrelation of DEP and ROT in the membrane dispersion range for a spherical model at low external conductivity. The “trick” of ROT is to translate the temporal phase shift between the induced dipole moment and the external field observed in linear fields into a spatial phase shift by applying a rotating external field (Fig. 1, bottom). The spatial phase shift leads to a torque, which is given by the cross product of the induced dipole moment and the external field. Accordingly, ROT detects the frequency dependence of the 90°‐phase shift, i.e. the out‐of‐phase part of the induced dipole moment. Mathematically, the out‐of‐phase part can be described as the imaginary part, which is at a maximum when the frequency of the exciting field is in accordance with the relaxation time of the object's polarization.

#### 2.2 Characteristics of DEP and ROT spectra

**Figure 2:**
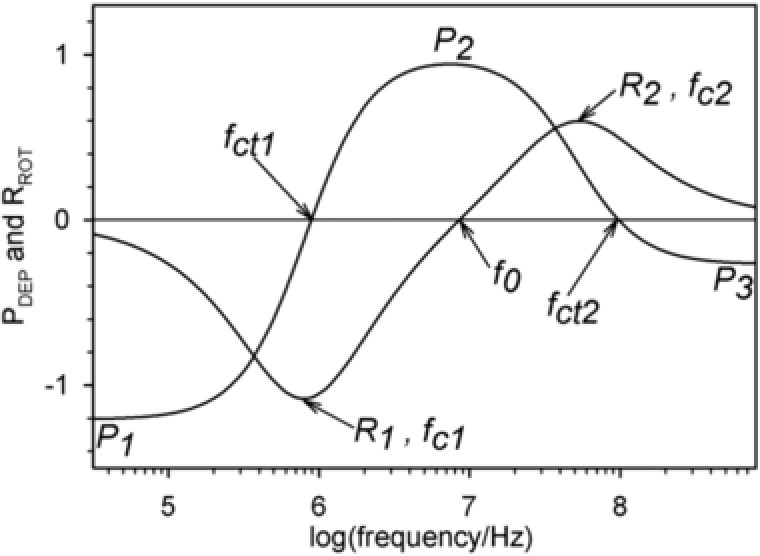
Typical $E_{0}^{2}$‐normalized DEP velocity (*P*_DEP_) and ROT speed (*R*_ROT_) spectra of a biological cell (cf. Eqs. (1) and (2)). The DEP spectrum is characterized by three velocity plateaus (*P*_1_, *P*_2_, *P*_3_) and two critical frequencies (*f*_ct1_, *f*_ct2_), while the related ROT spectrum is characterized by two rotation peaks (R_1_, R_2_) around the characteristic frequencies (*f*_c1_, *f*_c2_). Rotation ceases at *f*_0_.

Generally, the relation of DEP and ROT spectra is guided by Kramers–Kronig's relation 46, 49. Figure 2 shows typical interrelated spectra for the single‐shell model. The anti‐field ROT peak is located at the half‐value of the DEP dispersion from a negative to a positive plateau. At the peak, the out‐of‐phase part of the induced dipole moment is at a maximum and the angle between the external field and the induced dipole moment is 45° (Fig. 1, bottom). The capacitive membrane dispersion mediates the transformation of the cell polarization, which is governed by the nonconducting membrane below the dispersion to a polarization governed by the ionic conductivity relations of the bulk media. While the membrane dispersion mediates the transition between the force plateaus *P*_1_ and *P*_2_, resulting in the first, anti‐field ROT‐peak *R*_1_ around the characteristic frequency *f*_c1_, the second dispersion results from the superseding of the bulk conductivity‐related polarization by a bulk permittivity‐related polarization. This dispersion leads from force plateau *P*_2_ to *P*_3_ while giving rise to the second, co‐field ROT peak *R*_2_ around the characteristic frequency *f*_c2_. At low external and membrane conductivities, the plateaus *P*_1_ and *P*_3_ are usually negative, while *P*_2_ is positive for biological cells with intact membranes 1, 26, 27, 42. At the critical frequencies *f*_ct1_ and *f*_ct2_, the DEP force vanishes, i.e. the magnitude of the in‐phase (real) part of the induced dipole moment is zero. Figure 2 shows that the transitions in the in‐phase part of the induced dipole moment are complemented by transitions in the out‐of‐phase parts.

Because DEP and ROT are based on the interaction of the induced dipole moment with the external field, both effects are proportional to the square of the field strength. For a better comparability of the experiments, ROT and DEP data are usually normalized to the square of the field strength ($E_{0}^{2}$). The DEP velocity (*P*_DEP_) and ROT speed (*R*_ROT_) spectra of spherical or oriented ellipsoidal single‐shell objects can be described phenomenologically by simple Lorentzian terms. In DEP, the two transitions between the three plateaus *P*_1_, *P*_2_, and *P*_3_ around the characteristic frequencies *f*_c1_ and *f*_c2_ are described by:

(1) $$\begin{array}{ccc} P_{DEP} & = & \frac{f_{fric}^{DEP}}{E_{0}^{2}}v_{c}\left(f\right)=P_{3}+\frac{P_{1}-P_{2}}{1+\;{\left(f/f_{c1}\right)}^{2}}\\ & & +\frac{P_{2}-P_{3}}{1+\;{\left(f/f_{c2}\right)}^{2}}. \end{array}$$

The transitions correspond to the ROT peaks *R*_1_ and *R*_2_:

(2) $$\begin{array}{ccc} R_{ROT} & = & \frac{f_{fric}^{ROT}}{E_{0}^{2}}\omega_{c}\left(f\right)=\frac{f_{fric}^{ROT}2\pi }{E_{0}^{2}T_{c}\left(f\right)}=\;\frac{2\;R_{1}f/f_{c1}}{1+\;{\left(f/f_{c1}\right)}^{2}}\\ & & +\frac{2\;R_{2\;}f/f_{c2}}{1+\;{\left(f/f_{c2}\right)}^{2}}, \end{array}$$

where *f*, $v_{c}\left(f\right)\;$, $\omega_{c}\left(f\right)$, and $T_{c}\left(f\right)$ stand for the field frequency, the frequency‐dependent DEP velocity (in m/s), the angular ROT speed (in rad/s), and the time for one revolution of the object, respectively.$\;f_{fric}^{DEP}$ and $f_{fric}^{ROT}$ are friction coefficients, which depend on the objects’ size, shape, and orientation, as well as the distance to the chamber surfaces affecting the drag forces. Another problem is that the force‐coupling efficiencies between the field‐forces acting on the induced polarization charges and the object's interfaces are not well‐defined (compare to the shear plane in electrophoresis). As a result, relative changes in the force and torque effects (*R*1/*R*2 for example) can be registered more precisely than their absolute values. Critical and characteristic frequencies can be detected with compensation methods 26, 37, 50.

## 3 Theory

### 3.1 The external field

The external AC field can generally be written as:

(3) $$E=\;\left(\begin{array}{c} E_{x}\\ E_{y}\\ E_{z} \end{array}\right)=\;E_{o}e^{j\omega t}\left(\begin{array}{c} e_{x}\\ e_{y}\\ e_{z} \end{array}\right).$$

With *E*_o_ and *t* standing for field amplitude and time, respectively. The components of the field vector $E_{x},\;E_{y},\;E_{z}$ are oriented in the directions of the orthonormal base system *i*, *j*, and *k*. Different values of the *e*‐components describe different amplitudes of the field components, i.e. different field properties. In the standard situation, the semiaxes *a*, *b*, and *c* of the ellipsoidal object should be oriented in parallel to *i*, *j*, and *k*, respectively.

### 3.2 The induced dipole moment

In time‐harmonic fields, the dipole moment of cell‐size objects can be modeled using the electro‐quasistatic approximation if the dimensions of the measuring chamber are small with respect to the wavelength of the field. For the microchip chamber used here, the approximation is applicable up to the low GHz‐range. Moreover, for objects that are small in comparison to the characteristic distances of field strength variations the dipole approximation can be used to describe AC electrokinetic effects.

In component notation, the induced dipole moment of an object of the general ellipsoidal shape with semiaxes *a*, *b*, andc is 2, 40, 42, 44, 45:

(4) $$m=\;\left(m^{a}\;\;m^{b}\;\;m^{c}\right)=\;\varepsilon_{0}\varepsilon_{e}V\;\left(f_{CM}^{a}\;\;f_{CM}^{b}\;\;f_{CM}^{c}\right)\;E_{0},$$

where $\varepsilon_{e}$, ε_0_, V=4πabc/3, and *E*_0_ stand for the external and vacuum permittivity, the object's volume and the external field. $f_{CM}^{a}$ is the complex $f_{CM}$ in the direction of semiaxis *a*, i.e. the frequency‐dependent *a*‐component of the induced dipole moment. It is given by its real (superscript ℜ) and imaginary (superscript ℑ) parts, which are in‐phase and out‐of‐phase with the inducing field component:

(5) $$f_{CM}^{a}=\;f_{CM}^{aℜ}+\;jf_{CM}^{aℑ}.$$

### 3.3 Depolarizing factor and influential radius

The depolarizing factors of ellipsoidal objects are defined along the three principle axes 51, 52, 53, 54. Their sum is always unity:

(6) $$n^{a}+n^{b}+n^{c}\;=\;1.$$

Converting the depolarizing factors into influential radii 3, 39, 55. Along semiaxes *a*, the relative influential radius is:

(7) $$a_{inf}^{rel}=\frac{a_{inf}}{a}=\frac{1}{1-n^{a}}.$$

Analogous expressions are valid for the other two axes. $a_{inf}^{rel}$ is the maximum amplification factor for the local field, which is observed for vacuum objects 3. Accordingly, the influential radii along each principle semiaxis are the limiting distances from the object's respective symmetry planes to those equipotential planes that are just touching the respective poles of a vacuum object of identical shape.

### 3.4 The Clausius–Mossotti factor (***f***_***CM***_)

Influential radii permit the easy separation of the electrical problem from the geometric problem 3. For this, the complex $f_{CM}$ is expressed by the pole potentials 42. Along semiaxis *a*, the $f_{CM}$ is expressed by the potentials at the site of pole *a* in the presence (subscript *c*) and the absence (subscript 0) of the object:

(8) $$f_{CM}^{a}=\frac{1}{n^{a}}\;\left(\frac{\Psi_{0}^{a}-\;\Psi_{c}^{a}}{\Psi_{0}^{a}}\right).$$

The pole potentials are described using a special finite element approach, which assumes chains of finite elements along each principal semiaxes 42. For single‐shell objects, the three elements per axis form voltage dividers between the reference potential of 0 V, which is assumed at the symmetry planes of the object and the maximum possible pole potentials $a_{inf}E_{0}^{a}$, $b_{inf}E_{0}^{b}$, and $c_{inf}E_{0}^{c}$, which are obtained at sites in the external medium along the three semiaxes in the absence of the object. Along each axis, the electric properties of the three elements are determined by the geometry and the electric properties of the internal, membrane, and external media (subscripts *i*, *m*, and *e*). The actual potential $\Psi_{c}^{a}$ is given by electrically dividing the maximum possible pole potential $a_{inf}E_{0}^{a}$:

(9) $$\Psi_{c}^{a}=\;\frac{Z_{i}^{a}+\;Z_{m}^{a}}{Z_{i}^{a}+\;Z_{m}^{a}+\;Z_{e}^{a}}\;a_{inf}E_{0}^{a}.$$

The impedance of each geometric element is given by its actual length and an arbitrary cross sectional area *A*, which is assumed to be equal for all elements. For element *q* of length $l_{q}$ the impedance is:

(10) $$Z_{q}=\;\frac{1\;\;\;\;}{\sigma_{q}+j\omega \varepsilon_{q}\varepsilon_{o}}\;\frac{l_{q}}{A},$$

where *j*, and *ω* stand for $\sqrt{-1}$ and the circular frequency. $\sigma_{q}$ and $\varepsilon_{q}$ are the specific conductivity and the relative permittivity of the considered medium. The cross sectional areas of all elements are cancelling out in Eq. (9). The membrane thickness was neglected for the lengths of the internal elements. The lengths of the external elements are given by the differences of influential radii and the semiaxis lengths.

Using Eq. (9), Eq. (8) reads:

(11) $$f_{CM}^{a}=\frac{a_{inf}}{a_{inf}-\;a}\;\left(1-\;\frac{Z_{i}^{a}+\;Z_{m}^{a}}{Z_{i}^{a}+\;Z_{m}^{a}+\;Z_{e}^{a}}\;\frac{a_{inf}}{a}\right).$$

Analog expressions hold for the other two semiaxes. The equation is mathematically identical to the Laplace‐solution for homogeneous ellipsoidal objects when the membrane element $Z_{m}^{a}$ is neglected.

### 3.5 Area‐specific shell (membrane) properties

Clearly, the membrane thickness ($l_{q}=d$) is poorly defined for biological cells. Introducing area‐specific membrane conductivity ($g_{m}=\frac{\sigma_{m}}{d}$) and capacitance ($C_{m}=\frac{C}{A}=\frac{\varepsilon_{m}\varepsilon_{0}}{d}$) allows us to circumvent the assumption of a certain membrane thickness *d* 32. The membrane impedance along all three semiaxes reads:

(12) $$Z_{m}^{a}=Z_{m}^{b}=Z_{m}^{c}=\frac{1}{\sigma_{m}+j\omega \varepsilon_{m}\varepsilon_{0}}\frac{d}{A}=\frac{1}{\left(g_{m}+j\omega \;C_{m}\right)A}.$$

For three‐axial objects, the assumption of the same membrane properties along each of the three principal semiaxis is equivalent to the assumption of different model geometries for the field components oriented along each semiaxis. The reason is that the fundament of the influential radius approach is Laplace's equation, the solution of which requires a confocal geometry for the media interfaces 23, 31, 38, 44. The finite element approach combines three different model geometries for the three field components, which are oriented along the three principle axes. This ensures the assumption of the “correct” membrane thickness for each field component pointing at the respective pole of the ellipsoid. Because the membrane area around this pole contributes most to the interaction of the cell with the respective field component, the correspondence of the finite element approach with the biological situation is probably better than that of the classical Laplace model.

### 3.6 Forces and torques on ellipsoidal objects

Different AC‐electrokinetic phenomena can be observed when ellipsoidal objects are exposed to homogeneous, inhomogeneous, and rotating fields or the dipole fields of neighboring objects. The time‐averaged force 〈F〉 can be expressed by the real part of the scalar product of the induced dipole moment *m* and the gradient of the complex conjugate of the external field $E^{*}$ 39:

(13) $$\left\langle F\right\rangle =\frac{1}{2}ℜ\left[m\nabla E^{*}\right].$$

The time‐averaged torque 〈*N*〉 is given by the cross product of induced dipole moment and conjugated field in circular polarized fields:

(14) $$\left\langle N\right\rangle =\frac{1}{2}ℜ\left[m\times \;E^{*}\right].$$

The mutual attraction of two or more oriented objects of similar properties is driven by the induced dipole–dipole interactions, subsequently leading to pearl‐chain formation or aggregation. The effect can be seen as the mutual DEP of two objects in the inhomogeneous fields induced by their presences. Applying Coulomb's law to the dipole–dipole interaction of two objects with the same properties, i.e. the interaction of their four centers of charge, we obtain:

(15) $$\left\langle F_{i}\right\rangle =\;\frac{\varepsilon_{0}\varepsilon_{e}\;V^{2}}{4\pi r^{4}}{\left|f_{CM}^{a}\right|}^{2}E_{0}^{2}i,$$

for distances that are large with respect to the objects’ radii, where *r* stands for the distance of the centers of the two objects. Equation (15) assumes that the connecting line between the centers of the objects and their *a*‐axes are aligned with the field.

Neglecting thermal movements, the weakly inhomogeneous DEP fields will orient freely suspended, ellipsoidal objects. Accordingly, always one of the main axes will be oriented in field direction and the frequency‐dependent reorientations lead to discontinuous DEP spectra. For the orientation of semiaxis *a* in *x*‐direction of the external field, the DEP force is obtained after introducing Eq. (5) into Eq. (13):

(16) $$\left\langle F_{i}\right\rangle =\;\frac{1}{2}\;\varepsilon_{0}\varepsilon_{e}Vℜ\;\left[\left(f_{CM}^{aℜ}+jf_{CM}^{aℑ}\right)\;E_{x}\nabla E_{x}^{*}\right]i.$$

A weakly inhomogeneous field can be approximated by $e_{x\;}=1+\gamma x$ with $e_{y}=\;e_{z}=0$ 39. The parameter γ describes the field inhomogeneity. We get:

(17) $$\left\langle F_{i}\right\rangle =\;\varepsilon_{0}\varepsilon_{e}Vf_{CM}^{aℜ}E_{0}^{2}\frac{\gamma }{2}i\;.$$

For γ = 0, the external field is homogeneous, the DEP‐force vanishes, and Eq. (17) describes the electro‐deformation effect 56, 57.

### 3.7 Electro‐orientation

After introducing Eqs. (3) and (4) into Eq. (14), we get the time‐averaged torque 〈*N*〉 in component notation:

(18) $$\left\langle N\right\rangle =\;\frac{1}{2}\;ℜ\;\left(\begin{array}{ccc} m^{b}E_{z} & -\; & m^{c}E_{y}\\ m^{c}E_{x} & - & m^{a}E_{z}\\ m^{a}E_{y} & - & m^{b}E_{x} \end{array}\right).$$

For $e_{x}=\;e_{y}=\;e_{z}=1$ (Eq. (3)) the field is linear polarized. For an orientation of the ellipsoidal object with the semiaxes, a,b, and *c* in parallel to the vectors of the base system, the components of the external field along all semiaxes are equal and the induced torques around the three axes of the ellipsoid can be compared. Eq. (18) can be simplified to:

(19) $$\left\langle N\right\rangle \;=\;\frac{1}{2}\varepsilon_{e}\varepsilon_{0}VE_{0}^{2}\;\left(\begin{array}{ccc} f_{CM}^{bℜ} & - & f_{CM}^{cℜ}\\ f_{CM}^{cℜ} & - & f_{CM}^{aℜ}\\ f_{CM}^{aℜ} & - & f_{CM}^{bℜ} \end{array}\right).$$

The axis of the maximum real $f_{CM}$ part is oriented in the field direction. Even though the torque also vanishes for an orientation of the “wrong” axis in parallel to the field, this orientation is instable.

### 3.8 Electrorotation (ROT)

ROT is usually investigated in circular, steadily rotating, fields. A field rotating in the *x‐y* plane is obtained from Eq. (3) for $e_{x}=1$, $e_{y}=j,$ and $e_{z}=0$:

(20) $$E_{y}\;=\;jE_{x}\;E_{y}^{*}\;=\;-jE_{x}^{*}.$$

As considered below, such a field will discriminate against the axis with the lowest $f_{CM}$ and orient, for example semiaxis *c* perpendicular to the field. Accordingly, the *c*‐component of the induced dipole moment will vanish and axes *a* und *b* will experience the same field magnitudes. From Eq. (18) we obtain:

(21) $$\begin{array}{ccc} \left\langle N_{k}\right\rangle & = & \;\frac{1}{2}\varepsilon_{0}\varepsilon_{e}V\\ & & \;ℜ\left[\left(f_{CM}^{aℜ}+\;jf_{CM}^{aℑ}\right)E_{x}E_{y}^{*}-\;\left(f_{CM}^{bℜ}+\;jf_{CM}^{bℑ}\right)E_{y}E_{x}^{*}\right]k, \end{array}$$

which can be reduced to:

(22) $$\left\langle N_{k}\right\rangle \;=\;\varepsilon_{0}\varepsilon_{e}VE_{0\;}^{2}\frac{f_{CM}^{aℑ}+\;f_{CM}^{bℑ}}{2}\;k\;=\;\varepsilon_{0}\varepsilon_{e}VE_{0\;}^{2}f_{CM}^{abℑ}k,$$

with $\frac{f_{CM}^{aℑ}+\;f_{CM}^{bℑ}}{2}\;=\;f_{CM}^{abℑ}$ the equation shows that only the out‐of‐phase components of the induced dipole moment contribute to the torque. For the ellipsoidal objects, the three possible orientations lead to three different pairs of $f_{CM}$ components, which may be oriented in the field plane. Theoretically, these orientations lead to three different ROT spectra. Experimentally, reorientation will lead to a discontinuous ROT spectrum for freely suspended objects. For spheroidal or spherical objects Eq. (22) can further be simplified.

### 3.9 Characteristic equations for DEP and ROT spectra

In order to simplify the data interpretation, characteristic equations for certain features of the DEP and ROT spectra can be used. A complete set of the characteristic DEP and ROT equations for spherical objects was first derived by us 27. The set was later improved using the finite element approach and expanded to ellipsoidal objects 42.

For an object, oriented with semiaxis *a* in field direction, the DEP plateaus are given by:

(23) $$P_{1}^{a}=a_{inf}^{rel}\frac{ag\left(\sigma_{i}-\sigma_{e}\right)-\sigma_{i}\sigma_{e}}{ag_{m}\left(\left(a_{inf}^{rel}-1\right)\sigma_{i}+\sigma_{e}\right)+\sigma_{i}\sigma_{e}},$$

(24) $$P_{2}^{a}=a_{inf}^{rel}\frac{\sigma_{i}-\sigma_{e}}{\left(a_{inf}^{rel}-1\right)\sigma_{i}+\sigma_{e}},$$

(25) $$P_{3}^{a}=a_{inf}^{rel}\frac{\varepsilon_{i}-\varepsilon_{e}}{\left(a_{inf}^{rel}-1\right)\varepsilon_{i}+\varepsilon_{e}}.$$

The critical frequencies are:

(26) $$f_{ct1}^{a}\;=\;\frac{1}{2\pi aC_{m}}\sqrt{\frac{{\;\left(\;\sigma_{i}\;\sigma_{e}\;\right)}^{2}+\;\sigma_{i}\;\sigma_{e}ag_{m}\left(\;\left(2\;-\;a_{inf}^{rel}\;\right)\;\sigma_{i}\;-\;2\;\sigma_{e}\;\right)}{\;\left(\;\sigma_{i}-\;\sigma_{e}\;\right)\;\left(\;\left(a_{inf}^{rel}\;-\;1\;\right)\;\sigma_{i}\;+\;\;\sigma_{e}\;\right)}\;-\;{\;\left(ag_{m}\;\right)}^{2}},$$

(27) $$f_{ct2}^{a}=\frac{1}{2\pi \varepsilon_{0}}\sqrt{\frac{\left(\sigma_{i}-\sigma_{e}\right)\;\left(\left(a_{inf}^{rel}-1\right)\sigma_{i}+\sigma_{e}\right)}{\left(\varepsilon_{e}-\varepsilon_{i}\right)\left(\varepsilon_{e}+\left(a_{inf}^{rel}-1\right)\varepsilon_{i}\right)}\;\;}.$$

The derivation of comparably simple characteristic equations for the ROT spectra of three axial objects is not possible because always two axes are exposed to the circular polarized field. The different frequency‐dependencies of the $f_{CM}$ along these axes lead to mixed torque spectra (Eq. (22)), which deviate from the simple Lorentzian shape (Eq. (2)). Characteristic ROT equations can be derived for spherical objects (a=b=c=r) and spheroidal objects (a=b=r≠c) rotating around their symmetry axis 42:

(28) $$\begin{array}{ccc} R_{1}^{r}\;\; & = & \;-{r_{inf}^{rel}}^{2}\;\\ & & \frac{{\sigma_{i}}^{2}\sigma_{e}}{2\left(\left(r_{inf}^{rel}\;-\;1\right)\sigma_{i}\;+\;\sigma_{e}\right)\left(rg_{m}\left(\left(r_{inf}^{rel}\;-\;1\right)\sigma_{i}\;+\;\sigma_{e}\right)+\sigma_{i}\sigma_{e}\right)}, \end{array}$$

(29) $$R_{2}^{r}={r_{inf}^{rel}}^{2}\;\frac{\sigma_{i}\varepsilon_{e}-\sigma_{e}\varepsilon_{i}}{2\left(\left(r_{inf}^{rel}-1\right)\sigma_{i}+\sigma_{e}\right)\left(\left(r_{inf}^{rel}-1\right)\varepsilon_{i}+\varepsilon_{e}\right)},$$

(30) $$f_{c1}^{r}=\frac{1}{2\pi rC_{m}}\frac{\sigma_{i}\sigma_{e}}{\left(r_{inf}^{rel}-1\right)\sigma_{i}+\sigma_{e}}+rg_{m}\;,$$

(31) $$f_{c2}^{r}=\frac{1}{2\pi \varepsilon_{0}}\frac{\sigma_{e}+\;\left(r_{inf}^{rel}-1\right)\sigma_{i}}{\varepsilon_{e}+\left(r_{inf}^{rel}-1\right)\varepsilon_{i}}\;,$$

(32) $$f_{0}^{r}=\frac{1}{2\pi }\sqrt{\frac{\varepsilon_{0}rg_{m}^{2}\left(\varepsilon_{e}\sigma_{i}-\varepsilon_{i}\sigma_{e}\right)+\sigma_{i}^{2}\left(\varepsilon_{0}\varepsilon_{e}g_{m}-C_{m}\sigma_{e}\right)}{\varepsilon_{0}rC_{m}^{2}\left(\varepsilon_{e}\sigma_{i}-\varepsilon_{i}\sigma_{e}\right)+\varepsilon_{0}^{2}\varepsilon_{i}^{2}\left(\varepsilon_{0}\varepsilon_{e}g{}_{m}-C_{m}\sigma_{e}\right)}\;\;},$$

The above equations can be used to plot the characteristic parameters of DEP and ROT in phase diagram‐like planes, to interpret the time dependence of cellular parameters for example 26, 33, 37, 50.

## 4 Model parameters

The single‐shell ellipsoidal model used here approximates to the three‐axial shape of the CRBCs (Table 1) 58.

**Table 1:** Axis‐dependent parameter sets of the single‐shell ellipsoidal model. The influential radii were calculated as described in 39

Parameter	Single‐shell ellipsoid
Semiaxes (influential radii)	a: 7.70 μm (a_inf_: 8.66 μm)
	b: 4.00 μm (b_inf_: 5.47 μm)
	c: 1.85 μm (c_inf_: 4.87 μm)
Relative permittivity of cytoplasm, ε_i_	a: 70
	b: 60
	c: 50
Cell‐membrane capacitance, C_m_	10 mF/m²
Cell‐membrane conductivity, g_m_	3500 S/m²
Cytoplasmic conductivity, σ_i_	a: 0.36 S/m
	b: 0.36 S/m
	c: 0.36 S/m
Relative external permittivity, ε_e_	78.5

The cytoplasmic conductivity of 0.36 S/m and the specific membrane capacitance of 10 mF/m^2^ were assumed in accordance with a manuscript in preparation and experimental results on CRBCs already published 48, 58.

## 5 Results and discussion

### 5.1 Linear field orientation (LFO) and circular field orientation (CFO)

**Figure 3:**
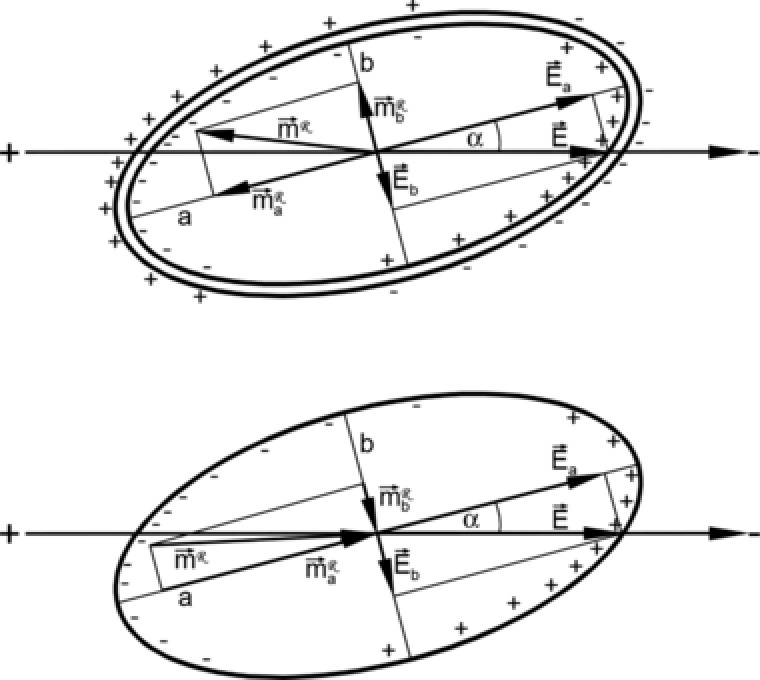
LFO of the single‐shell ellipsoidal model (Table 1) at an external conductivity of 0.15 S/m. The scheme shows the relations of the components $\vec{E_{a}}$ and $\vec{E_{b}}$ of the external field $\vec{E}$, the components of the induced dipole moment $\vec{m_{a}}$ and $\vec{m_{b}}$, and the influenced charges at 100 kHz (top) and 10 MHz (bottom), below and above the membrane dispersion, respectively. Charges and vector orientations represent the situation during one half‐cycle of the field. During the other half‐cycle, all the charge signs and vector orientations are reversed, resulting in the same torques. The angle α represents the initial misalignment between a and the external field.

Electro‐orientation spectra comprise two kinds of information: the frequencies of reorientation and the oriented axis for the frequency bands within the turnover frequencies 2, 18, 36, 55, 59. Figure 3 gives a schematic explanation for the linear field orientation (LFO) torques leading to the orientation of the same axis of a single‐shell ellipsoidal object at frequencies below and above the membrane dispersion, i.e. axis *a* will be oriented even though the polarizability of the object may be lower or higher than that of the external medium, respectively 55.

The generated torques can be explained using the vector components of the external field and the induced dipole moment as well as the induced charges. At frequencies below the membrane dispersion, the influenced charges around the poles are “pushed” by the external field aligning the axis with the field, which is “pushed” weakest. At frequencies above the membrane dispersion, the orientation of the induced dipole moment is inverted and the influenced charges around the poles are “pulled” by the external field aligning the axis with the field, which is “pulled” strongest. As a rule, the axis with the highest real $f_{CM}$ component is oriented in the field direction in LFO (Eq. (19)).

The different $f_{CM}$ values along the semiaxes result in a ratio of the components of the induced dipole moment of semiaxes *a* and b, which differs from the ratio of the field components. At both frequencies, the interaction of the resulting dipole moments with the external field generates torques that align semiaxis *a* with the external field. In circular field orientation (CFO), the two axes with the highest $f_{CM}$ are oriented in the field plane. Accordingly, CFO discriminates against the axis with the lowest $f_{CM}$, which will be oriented perpendicular to the plane.

**Figure 4:**
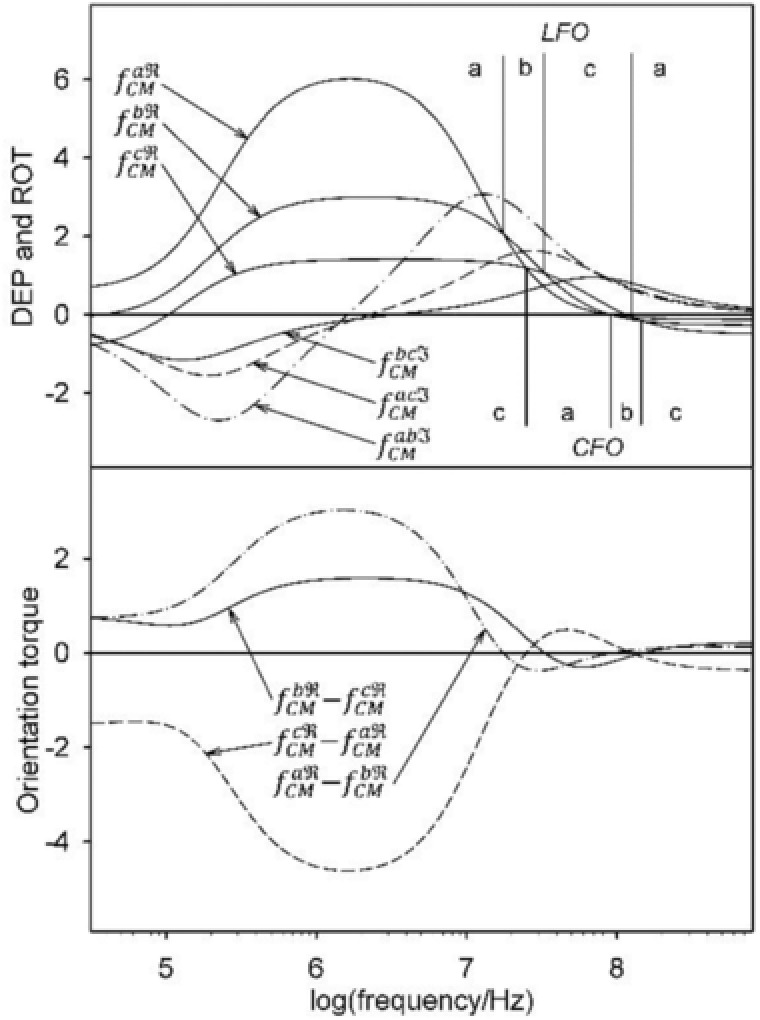
Top: $Real(f_{CM})$ spectra and combined *Imag* $\left(f_{CM}\right)$ spectra (see Eq. (22)) at an external conductivity of 0.015 S/m. Bottom: the difference spectra govern LFO and CFO according to Eq. (19). Zeros in the difference spectra mark the reorientation frequencies in either LFO or CFO. Experimental DEP spectra for freely suspended objects are obtained by combining the branches forming the top edge of the $Real(f_{CM})$ spectra. The bottom edge determines the semiaxes, which is oriented perpendicular to the field plane in CFO and ROT. The torque generation occurs around the other two axes. The experimentally observed reorientation results in broken ROT spectra. For details see Fig. 5.

In LFO and CFO, reorientations are observed only at frequencies above 10 MHz. While three different orientations are observed at an external conductivity of 0.015 S/m (Fig. 4), only two orientations are observed at 0.15 S/m (Fig. 6). At 0.3 S/m and higher conductivities, the orientations of axes *a* (LFO) and c (CFO) are stable (Figs. 6B and C). The torque spectra (Eq. (19), bottom diagrams in Figs. 4 and 6) suggest frequency ranges of strong and weak orientation. The complexity of the torque spectra and the experimental scatter in the individual object properties explains why orientation spectra are harder to interpret than DEP and ROT spectra 2, 60, 61, 62, 63.

**Figure 5:**
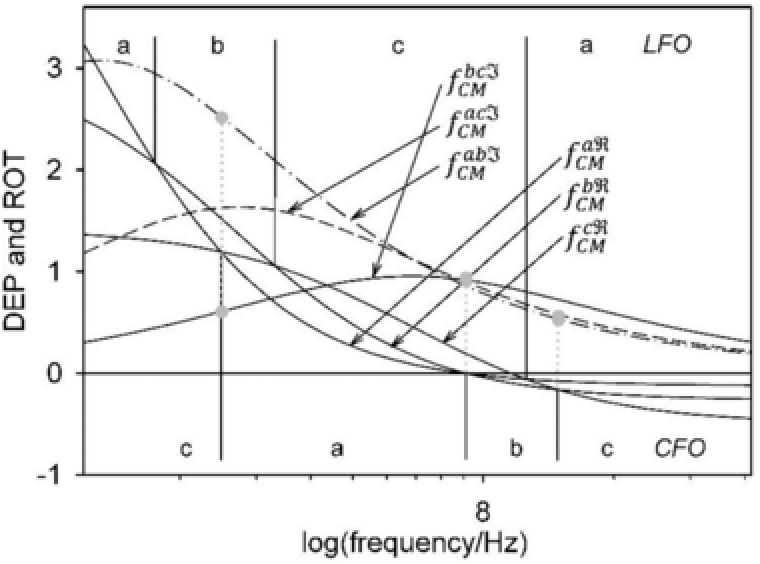
Zoom of Fig. 4 for the frequency range where reorientations occur. The experimental spectra are combined of different branches of the theoretical spectra, leading to inflection points and breaks in the DEP and ROT spectra, respectively. For increasing frequencies, the resulting ROT spectrum combines branches of the three ROT curves in the dash‐dotted – solid – dashed ‐ dash‐dotted sequence. The breaks in the resulting spectrum are marked by vertical, grey dotted lines.

**Figure 6:**
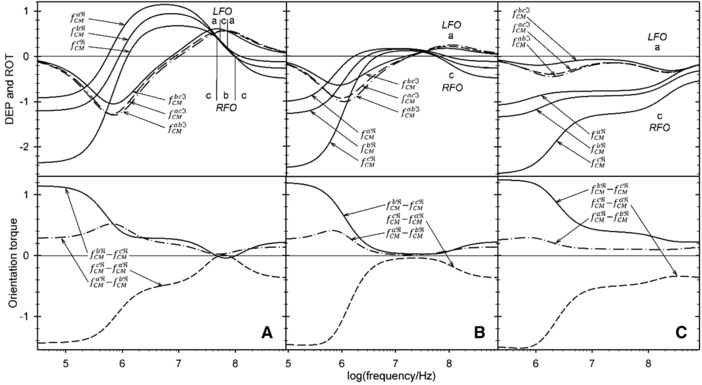
Spectra at external conductivities of 0.15(A), 0.3(B), and 1.2 S/m(C). For an explanation, see Fig. 4.

### 5.2 Dielectrophoresis (DEP), electrorotation (ROT) and orientation torque spectra

The top panel of Fig. 4 presents $Real(f_{CM})$ spectra and combined $Imag(f_{CM})$ *spectra* at an external conductivity of 0.015 S/m. The spectra govern DEP and ROT according to Eqs. (17) and (22), respectively. The bottom panel displays spectra of the torques around the three principal axes according to Eq. (19). The spectra suggest a complex frequency dependence for the LFO and CFO torques. Every zero in one of the three torque functions, i.e. a vanishing torque around one axis, is related to a frequency‐dependent change in the axis of the maximum or minimum $f_{CM}$.

In the top panels, the $Real(f_{CM})$ branches are designated by the semiaxes, which exhibit maxima or minima of the $Real(f_{CM})$. For increasing frequencies, maxima and minima change in a−b−c−a and c−a−b−c sequences, respectively. The maxima forming the top edge of the $Real(f_{CM})$ spectra reflect the DEP spectrum, which will be experimentally observed for freely oriented objects, while the bottom edge determines the semiaxes being oriented in CFO. In ROT experiments, reorientation will lead to “broken” spectra.

Figure 6 summarizes the spectra for 0.15, 0.3, and 1.2 S/m.

### 5.3 Dielectrophoresis (DEP), linear field orientation (LFO), and circular field orientation (CFO)

**Figure 7:**
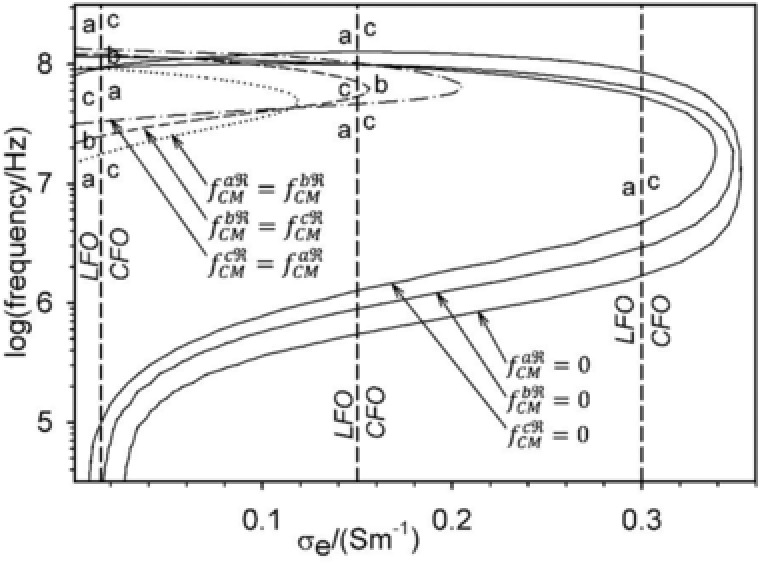
Critical DEP frequencies ($f_{CM}^{aℜ}=f_{CM}^{bℜ}=f_{CM}^{cℜ}=0$) and reorientation frequencies ($f_{CM}^{aℜ}=f_{CM}^{bℜ}$, $f_{CM}^{bℜ}=f_{CM}^{cℜ}$, and $f_{CM}^{aℜ}=f_{CM}^{cℜ}$) over external medium conductivity. The oriented axes for LFO and CFO are marked for the external conductivities of 0.015, 0.15, and 0.3 S/m used in Figs. 4, 5, 6 (vertical dashed lines). The reorientation curves limit areas of certain orientations in LFO and CFO and the critical frequencies of DEP.

The critical DEP frequencies over external conductivity can be obtained from complete DEP spectra (Figs. 4 and 6) or, directly from Eq. (26) ($f_{ct1}$, low frequency branch) and Eq. (27) ($f_{ct2}$, high frequency branch) for axis *a* ($f_{CM}^{aℜ}=0$), *b* ($f_{CM}^{bℜ}=0$), and *c* ($f_{CM}^{cℜ}=0$) being oriented in field direction (Fig. 7). The two branches for each orientation join at external conductivities above which the object's polarizability is lower than that of the external medium. Accordingly, this point is shifted towards higher external conductivities for higher internal conductivities.

The reorientation frequencies are determined by the conditions $f_{CM}^{aℜ}=f_{CM}^{bℜ}$, $f_{CM}^{bℜ}=f_{CM}^{cℜ}$, and $f_{CM}^{aℜ}=f_{CM}^{cℜ}$ (Eq. (19)). These conditions define the borderlines of areas of different maxima or minima of the $f_{CM}$ in a frequency‐over‐external‐conductivity plot (Fig. 7). Thus, the borderlines separate areas of certain orientations in LFO and CFO. When a line is crossed, the oriented axis changes, either in LFO or CFO.

The dashed vertical lines in Fig. 7 illustrate the LFO and CFO behavior considered for the external conductivities of 0.015, 0.15, and 0.3 S/m (compare to Figs. 4, 6A and B). At 0.015 S/m, the sequences of axes orientation are *a*‐*b*‐*c*‐*a* in LFO and *c*‐*a*‐*b*‐*c* in CFO when starting at low frequencies. At 0.15 S/m, the sequences are *a‐c‐a* in LFO and *c‐b‐c* in CFO. Outside the outermost circumference of the reorientation curves (“little noses”) the orientations are stable as marked for 0.3 S/m.

## 6 Concluding remarks

From Maxwell's equivalent body notion, it follows: (i) that the effective local field of the shelled object is constant, (ii) that the external field distributions of Maxwell's equivalent body and the shelled object are identical, and (iii) that the effective local field (and the induced dipole moment) can be obtained from the pole potentials. In consequence, measurements of the dipole moment do not principally permit to distinguish whether the frequency dependence of the dipole moment stems from internal object structures or from frequency‐dependent material properties 64.

When the object geometry and dielectric properties of the object's compartments are known, its induced dipole moment and all electrokinetic spectra and re‐orientation frequencies can be predicted unambiguously. Nevertheless, in most cases, finding a consistent set of geometric and dielectric parameters for biological cells or colloidal particles seems to be unrealistic. Accordingly, the reliable prediction of the spectra of other AC‐electrokinetic spectra based on the dielectric model obtained from measurements with a first method is usually impossible. Nevertheless, such a prediction is theoretically possible when the measuring points of DEP and ROT spectra are generated from the same model by the addition of artificial noise 46.

In summary, the search for consistent dielectric object properties will be more promising the greater the number of methods employed. There is hope that the combination of multiple AC‐electrokinetic methods may lead to a new kind of DSCS fingerprinting with a higher resolution for cell and particle structures 29, 34, 35, 65.

*The author has declared no conflict of interest*.
